# Online Learning for Wearable EEG-Based Emotion Classification

**DOI:** 10.3390/s23052387

**Published:** 2023-02-21

**Authors:** Sidratul Moontaha, Franziska Elisabeth Friederike Schumann, Bert Arnrich

**Affiliations:** Digital Health—Connected Healthcare, Hasso Plattner Institute, University of Potsdam, 14482 Potsdam, Germany

**Keywords:** online learning, real-time, emotion classification, AMIGOS dataset, wearable EEG (muse and neurosity crown), psychopy experiments

## Abstract

Giving emotional intelligence to machines can facilitate the early detection and prediction of mental diseases and symptoms. Electroencephalography (EEG)-based emotion recognition is widely applied because it measures electrical correlates directly from the brain rather than indirect measurement of other physiological responses initiated by the brain. Therefore, we used non-invasive and portable EEG sensors to develop a real-time emotion classification pipeline. The pipeline trains different binary classifiers for Valence and Arousal dimensions from an incoming EEG data stream achieving a 23.9% (Arousal) and 25.8% (Valence) higher F1-Score on the state-of-art AMIGOS dataset than previous work. Afterward, the pipeline was applied to the curated dataset from 15 participants using two consumer-grade EEG devices while watching 16 short emotional videos in a controlled environment. Mean F1-Scores of 87% (Arousal) and 82% (Valence) were achieved for an immediate label setting. Additionally, the pipeline proved to be fast enough to achieve predictions in real-time in a live scenario with delayed labels while continuously being updated. The significant discrepancy from the readily available labels on the classification scores leads to future work to include more data. Thereafter, the pipeline is ready to be used for real-time applications of emotion classification.

## 1. Introduction

Emotions play a crucial role in human communication and cognition, which makes comprehending them significant to understanding human behavior [1]. The field of affective computing strives to give emotional intelligence to machines that can recognize and interpret human affects [1,2], offering exciting possibilities for education, entertainment, and healthcare. Early detection and prediction of (mental) diseases or their symptoms can be facilitated, since specific emotional and affective states are often indicators thereof [3]. Moreover, long-term stress is one of today’s significant factors causing health problems, including high blood pressure, cardiac diseases, and anxiety [4]. Notably, some patients with epilepsy (PWE) report premonitory symptoms or auras as specific affective states, stress, or mood changes, enabling them to predict an oncoming seizure [5]. This association of premonitory symptoms and seizure counts has been analyzed and validated from patient-reported diaries [6], and non-pharmacological interventions were proven to reduce the seizure rate [7]. However, many PWE cannot consistently identify their prodromal symptoms, and many do not perceive prodromes [8], emphasizing the necessity of objective prediction of epileptic seizures. In a previous work, the authors proposed developing a system to predict seizures by continuously monitoring their affective states [9]. Therefore, identifying pre-ictal states by measuring and predicting affective states in real-time through neurophysiological data could aid in finding pre-emptive therapies for PWE. That would be incredibly beneficial, especially to people with drug-resistant epilepsy, and would improve their quality of life [3,8]. Consequently, emotion detection in this paper is motivated by the idea that allowing computers to perceive and understand human emotions could improve human–computer interactions (HCI) and enhance their ability to make decisions by adapting their reactions accordingly.

Since emotional reactions are seemingly subjective experiences, neurophysiological biomarkers, such as heart rate, respiration, or brain activity [10,11], are inevitable. Additionally, for continuous monitoring of affective states and thus detecting or predicting stress-related events reliably, low-cost, consumer-grade devices rather than expensive and immobile hospital equipment would be more meaningful [12]. This is an important area of interest in cognitive science and affective computing, with use cases varying from designing brain–computer interfaces [13,14] to improving healthcare for patients suffering from neurological disorders [15,16]. Among these, electroencephalography (EEG) has proven to be an accurate and reliable modality without needing external annotation [17,18]. Since clinical EEG is the gold standard for epileptic seizure detection [19], utilizing EEG-based emotion classification in detection systems could potentially predict seizures by knowing the affective states. Moreover, with recent advancements in wearable technology, consumer-grade EEG devices have become more accessible and reliable, opening possibilities for countless real-life applications. Wearable EEG devices such as the *Emotiv EPOC* headset or the *Muse S* headband have become quite popular tools in emotion recognition [20,21,22]. The Muse S headband has also been used for event-related potential (ERP) research [12] and for the challenge of affect recognition in particular. More specifically, Muse S has already been used in experimental setups to obtain EEG data from which the mental state (relaxed/concentrated/neutral) [13] and the emotional state (using the valence-arousal space) [23] could be reliably inferred through the use of a properly trained classifier.

### 1.1. Problem Statement

A challenging but essential step to identifying stress-related events or improving HCI in real-life settings is to recognize changes in peoples’ affect by leveraging live data. The EEG-based emotion classifications mentioned in the literature have nearly exclusively been employed in traditional machine learning strategies, i.e., offline classifiers, and are often combined with complex data pre-processing techniques on static datasets [24,25]. Such cases expect the whole dataset, including labels, to be present for model training, unlike real application scenarios, where the data source is primarily a live data stream and classification results are required in real-time. Moreover, a live data stream from a non-stationary EEG data source presents challenges associated with, for example, the online arrival of data, the velocity of data, the data volume over time, and the dynamic nature of data [26]. The training and classification time for the offline models and the static behavior dealing with upcoming data streams makes real-time classification of affective states infeasible. Therefore, for live emotion classification, employing online learning by updating a pre-trained model continuously on new data is inevitable. Additionally, to ensure the usability of such a system in daily life, it is necessary to classify the data from a portable EEG device. Furthermore, the existing methodologies in the literature have primarily been developed and evaluated on curated EEG datasets, which need more reproducibility to apply to live data from real applications.

Regarding the mentioned problems, the primary goal of this paper is to answer the research question of how reliably an online classifier classifies emotion from state-of-the-art AMIGOS data [22] and how accurately the classifier can perform affective state prediction while curating EEG data from wearable EEG devices in live settings.

### 1.2. Key Contributions

Therefore, *firstly*, the key contribution of this paper is the establishment of a lightweight emotion classification pipeline that can classify a person’s affective state based on an incoming EEG data stream in real-time, efficiently enough to be used in real applications, e.g., for seizure prediction. The developed pipeline leverages online learning to train subject-specific models on data streams by implementing binary classifiers for the affect dimensions: *Valence* and *Arousal*. The pipeline is validated by streaming an existing dataset of established quality, AMIGOS, with better classification performance than state-of-the-art contributions.

*Secondly*, an experimental framework is developed, similar to the AMIGOS dataset, which can collect neurophysiological data from a wide range of commercially available EEG devices and show live prediction results of the subjects’ affective states even when labels arrive with a delay. Data from 15 participants were captured by using two consumer-grade EEG devices.

*Thirdly*, the most novel contribution of this paper is to validate the pipeline on the curated dataset by wearable EEG devices in the first experiment with consistent classification performance on the AMIGOS dataset. Following this, live emotion prediction was performed successfully on an incoming data stream in the second experiment with delayed incoming labels.

The curated data from the experiments and metadata are accessible to the designated researchers as per the participants’ consent. Therefore, the dataset is available upon request for scientific use via a contact form on Zenodo: https://doi.org/10.5281/zenodo.7398263 (accessed on 18 February 2023). The Python code for loading the dataset and implementations of the developed pipeline have been made available on GitHub: https://github.com/HPI-CH/EEGEMO (accessed on 18 February 2023). The next section will explain the related works, material, and methods utilized within this paper following the results and discussion sections.

### 1.3. Related Work

Recent review papers from Dadebayev et al. [24] and Suhaimi et al. [25] mention several articles on EEG-based emotion recognition using various affect dimension scales, EEG devices, machine learning algorithms, and performance matrices. However, only a few research works have mentioned *real-time* emotion classification. Müller et al. [27] proposed an online linear discriminate analysis (LDA) classifier by utilizing spectral features in the EEG data obtained from a brain–computer interface (BCI) device. However, while demonstrating the application of real-time arousal monitoring, the researchers used data from one participant to train an offline LDA classifier. Moreover, the data acquisition took place utilizing a 128-channel BCI, and applying the methodology to the data from wearable EEG devices was mentioned in their future work, which already falls into the scope of this paper.

Liu et al. [28,29] showed promising results on emotion recognition using the fractal dimension (FD) model on a 14-channel EEG headset. The applied methodology in real-time application was reflected as computer avatars demonstrating a person’s emotion based on live data. The fractal algorithm required 1024 samples at a time, obtained by the device’s buffer function. The buffer function may not be useful for general applications and EEG devices. A follow-up work was proposed by Lan et al. [30,31], analyzing the stable features of the previously mentioned application. Hou et al. [32] further developed a meter to visualize the intensity of the felt emotion. However, the mentioned live emotion classification was based on a static model and was not updated during prediction. We propose a general emotion classification pipeline that deals with a selection of consumer-grade EEG devices and general applications, which can be validated on the published dataset from the researchers mentioned above [33].

Additionally, Javaid et al. [34] reported a 25.44% higher accuracy while switching from eight to three wet electrodes of an openBCI Kit while quantifying four basic emotions using an FD threshold-based classifier similar to [28]. However, the authors also mentioned incorporating an Emotiv EPOC device in a second session of their proposed method, but the follow-up research still needs to be reported.

Sarno et al. [35] used power features in the K-nearest neighbor (KNN) classifier to train 14-channel EEG data offline and predict binary and tertiary emotion in an online stage. An evaluation only of accuracy was reported, which is compatible with our results, given that the F1-Score reported in our paper is relatively high. Moreover, our paper uses fewer electrodes and multiple algorithms and develops and publishes the data collection framework.

Very recently, Bajada et al. [36] built an emotion classification pipeline incorporating discrete wavelet transforms (DWT) features into a 3D convolutional neural network (3D CNN) and support vector machine (SVM) classifiers from pre-recorded and pre-trained data from the state-of-the-art DEAP [37] dataset. They proposed using the proposed algorithm for real-time monitoring because it maintains a high accuracy of up to 92%, reducing from 32 channels to 5 channels. Li et al. [38] addressed the challenge of when a model can see the data only once by leveraging cross-subject and cross-session data by implementing the *Fast Online Instance Transfer (FOIT)* algorithm. They validated their methodology on two state-of-the-art datasets: SEED [39] and SEED-IV [40]. However, these mentioned studies need to apply live incoming data streams similar to our approach, which pose challenges such as velocity, veracity, and concept drift. To the best of our knowledge, only Nandi et al. [41] have employed online learning to classify emotions from an EEG data stream from the DEAP dataset and proposed an application scenario in e-learning, but have yet to report undertaking any such live experiments. They compared the performance of different state-of-the-art online classifiers, such as adaptive random forest (ARF) [26] and Hoeffding adaptive tree (HAT) [42], against their own real-time emotion classification system (RECS) on the DEAP dataset.

Indeed, more research is needed on using online machine learning for emotion recognition.

Moreover, multi-modal labeled data for the classification of affective states have been made freely available through annotated affective databases, such as DEAP [37], DREAMER [43], ASCERTAIN [44], SAFE [33], and AMIGOS [22], which play a significant role in further enhancing the research of this field. They include diverse data from experimental setups using differing emotional stimuli such as music, videos, pictures, or cognitive load tasks in an isolated or social setting. Such databases enable the development and improvement of frameworks and model architectures with existing data of ensured quality. However, none of these published datasets include the data collection framework to be reused in curating the data from wearable EEG devices in live settings.

## 2. Materials and Methods

### 2.1. Dataset I: AMIGOS Dataset

**Figure 1:**
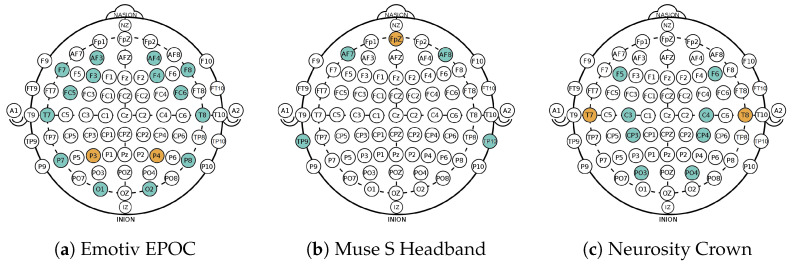
Different electrode positions according to the international 10–20 system of the EEG devices used in Dataset I (**a**) and in Dataset II and III (**b**,**c**). Sensor locations are marked in blue, references are in orange.

The developed emotion classification pipeline in this paper was evaluated on the state-of-art dataset for affect, personality, and mood research on individuals and groups (AMIGOS) published by Miranda-Correa et al. [22], which is further referred to as *Dataset I*. Upon following the data receiving protocol, all data from the AMIGOS dataset that are used in this work stem from short video individual experiments where 40 healthy participants (13 female) aged between 21 and 40 (mean age 28.3) were asked to watch 16 videos from defined movie clips. The EEG data were recorded using the Emotiv EPOC headset (https://www.emotiv.com/epoc-x/ (accessed on 20 February 2023)) with a sampling frequency of 128 Hz and a 14 bit resolution. This device records EEG data from 14 channels (AF3, F7, F3, FC5, T7, P7, O1, O2, P8, T8, FC6, F4, F8, and AF4) of the brain according to the 10–20 system as depicted in Figure 1a.

Additionally, Dataset I reports the Self-Assessment Manikin [45] with a scale from 1 to 9 as recording participants’ affect ratings of the dimensions valence, arousal, and dominance. The participants were also asked to rate their familiarity with the videos and whether they liked them, and had to select at least one option from a list of basic emotions felt after watching each video. However, only the obtained valence and arousal ratings were considered the ground truth for this paper. Furthermore, the participants answered the Positive and Negative Affect Schedules (PANAS) [46] questionnaire at the beginning and end of the experiment; only one overall calculated PANAS score is reported. Pre-processed data files were used for classification, where the EEG data were down-sampled to 128 Hz, averaged to a common reference, and a band pass filter was applied from 4.0–45.0 Hz as described in the description on the website (http://www.eecs.qmul.ac.uk/mmv/datasets/amigos/readme.html (accessed on 20 February 2023)). The files containing electrocardiogram (ECG) and galvanic skin response (GSR) data were removed for the analysis of this paper.

To validate the established pipeline in daily life and live setup, two different datasets with individual experimental protocols, named *Dataset II* and *Dataset III*, were curated with the description of participants, data acquisition, and experimental protocols mentioned in the following sections.

### 2.2. Participants

For Dataset II, eleven participants were recruited (six females and five males) between the ages of 25 and 42 (μ=29.27, σ=5.41 years). Data from two participants had to be discarded for further analysis. Dataset III was made with data from four participants (one female and three males) between the ages of 25 and 34 (μ=28.5, σ=3.5 years). People who were pregnant, older than 65 years, and had taken part in one of the experiments were excluded from participation. All participants had normal or corrected vision and reported no history of neurological or mental illnesses or head injuries.

### 2.3. Data Acquisition

**Figure 2:**
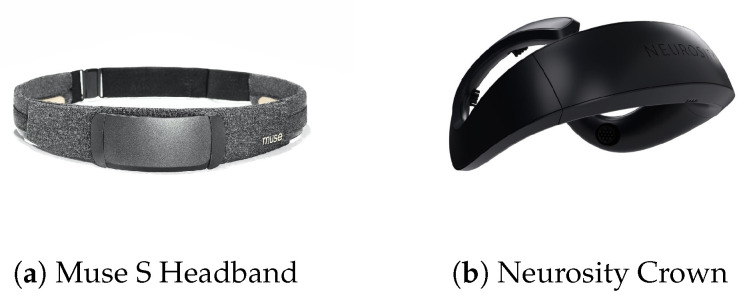
Two consumer-grade EEG devices with integrated electrodes used in the experiments.

**Hardware:** During the experiments, two consumer-grade devices, *Muse S Headband* Gen 1 (https://choosemuse.com/compare/ (accessed on 20 February 2023)) and *Neurosity Crown* (https://neurosity.co/crown (accessed on 20 February 2023)), were used to collect the EEG data from the participants, as depicted in Figure 2. Both devices operated with a sampling rate of 256 Hz and the EEG data were collected with four and eight channels, respectively.

According to the international10–20 system [47], the channels on the Muse S Headband correspond to AF7, AF8, TP9, and TP10 (see Figure 1b), with a reference electrode at Fpz [12]. The channel locations of Neurosity Crown are C3, C4, CP3, CP4, F5, F6, PO3, and PO4, with reference sensors located at T7 and T8, as shown in Figure 1c. Using the Mind Monitor App (https://mind-monitor.com/ (accessed on 20 February 2023)), the raw EEG data were streamed from Muse to a phone via Bluetooth. The app sends the data to a laptop via the open sound control (OSC) protocol and the python-osc library (https://pypi.org/project/python-osc/ (accessed on 20 February 2023)) on the receiving end. As the incoming data tuples from the Muse Monitor App did not include timestamps, they were added by the pipeline upon arrival of each sample. Similarly, the Crown uses the python-osc library to stream the raw EEG data to a laptop without enabling any pre-processing settings. In contrast to the Muse Headband, the Crown includes a timestamp when sending data.

**Figure 3:**
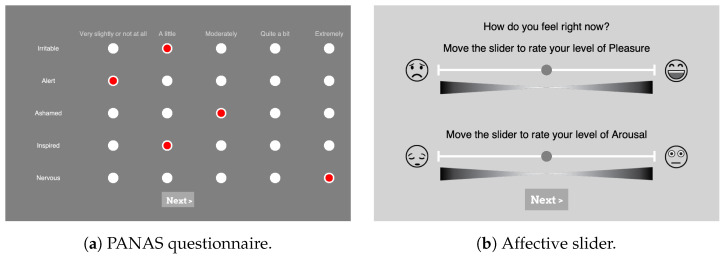
Screenshots from the PsychoPy [48] setup of self-assessment questions. (**a**) Partial PANAS questionnaire with five different levels represented by clickable radio buttons (in red) with the levels’ explanation on top, (**b**) AS for valence displayed on top and the slider for arousal on the bottom.

**Software:** In this paper, the experiment was implemented using the software PsychoPy (v 2021.2.3) [48] in a way that guided the participants through instructions, questionnaires, and stimuli. The participants were allowed to go at their own pace by clicking on the “Next” (“Weiter” in German) button, as shown in the screenshots of PsychoPy in Figure 3.

### 2.4. Stimuli Selection

Inducing specific emotional reactions is a challenge, even in a fully controlled experimental setting. Several datasets have tried to solve this issue with different modalities such as pictures [49,50,51], music [52,53], music videos [37,54,55], or combinations of them [56]. In this work, videos depicting short movie scenes were used as stimuli, based on the experimental setup of Dataset I [22]. Therefore, 16 short clips (51–150 s long, μ=86.7 s, σ=27.8 s) depicting scenes from 15 different movies were used for emotion elicitation. Twelve of these videos stem from the DECAF dataset [54], and four movie scenes were taken from the MAHNOB-HCI [55] dataset. According to Miranda-Correa et al., these specific clips were chosen because they “lay further to the origin of the scale” than all other tested videos. This means they represent the most extreme ratings in their respective category according to the labels provided by 72 volunteers. The labels were provided in the two-dimensional plane spanned by the two dimensions *Valence* and *Arousal* according to Russell’s circumplex model of affect [57]. Valence, the dimension describing one’s level of pleasure, ranges from sad (unpleasant and stressed) to happy (pleasant and content), and Arousal ranges from sleepy (bored and inactive) to excited (alert and active). Therefore, the four category labels are High Arousal, Low Valence (HALV); High Arousal, High Valence (HAHV); Low Arousal, High Valence (LAHV); and Low Arousal, Low Valence (LALV). The selected movie scenes described in Table 1 are balanced between each of the valence–arousal space quadrants (HVHA, HVLA, LVHA, and LVLA). The video ID 19 corresponded to a scene from the movie *Gandhi*, which differs from the AMIGOS dataset but falls into the same LALV quadrant.

### 2.5. Behavioral Data

**PANAS:** During the experiments, participants were asked to assess their baseline levels of affect in the PANAS scale. As depicted in Figure 3a, in total 20 questions (10 questions from each of the Positive Affect (PA) and Negative Affect (NA) dimensions) were answered using a 5-point Likert scale with the options ranging from “very slightly or not at all” (1) to “extremely” (5). To see if the participants’ moods generally changed over the course of the experiments, they were asked to answer the PANAS once at the beginning and once again at the end. For the German version of the PANAS questionnaire, the translation of Breyer and Bluemke [58] was used.
**Affect Self-Assessment:** The Affective Slider (AS) [59] was used in the experiment to capture participants’ emotional self-assessment after presenting each stimulus, as depicted in the screenshot in Figure 3b (https://github.com/albertobeta/AffectiveSlider (accessed on 20 February 2023)). The AS is a digital self-reporting tool composed of two slider controls for the quick assessment of pleasure and arousal. The two sliders show emoticons at their ends to represent the extreme points of their respective scales, i.e., unhappy/happy for pleasure (valence) and sleepy/wide awake for arousal. For the experiments, AS was designed in a continuous normalized scale with a step size of 0.01 (i.e., a resolution of 100), and the order of the two sliders was randomized each time.

**Familiarity:** The participants were asked to indicate their familiarity with each video on a discrete 5-point-scale ranging from “Have never seen this video before” (1) to “Know the video very well” (5). The PsychoPy slide with this question was always shown after the AS.

### 2.6. Dataset II

**Briefing Session:** In the beginning, each participant went through a pre-experimental briefing where the experimenter explained the study procedure and informed the participant of the experiment’s duration, i.e., two parts of approximately 20 min each with a small intermediate break. The participant then received and read the data information sheet, filled out the personal information sheet, and signed the consent to participate. Personal information included age, nationality, biological sex, handedness (left- or right-handed), education level, and neurological- or mental health-related problems. The documents and the study platform (i.e., PsychoPy) were provided according to the participant’s choice of study language between English and German. Afterward, the experimenter explained the three scales mentioned in and allowed the participant to accustom to the PsychoPy platform. This ensured the understanding of the different terms and scales used for the experiment without having to interrupt the experiment afterwards. The participant could refrain from participating at any moment during the experiment.
**Data Collection:** After the briefing, the experimenter put either the Muse headband or the Crown on the participant by a random choice. Putting headphones over the device, the participant was asked to refrain from strong movements, especially of the head. The experimenter then checked the incoming EEG data and let the participant begin the experiment. After greeting the participant with a welcome screen, a relaxation video was shown to the participant (https://www.youtube.com/watch?v=S6jCd2hSVKA (accessed on 20 February 2023)) for 3 min. They answered the PANAS questionnaire to rate their current mood and closed eyes for half a minute to get a baseline measure of EEG data. Afterwards, they were asked to initially rate the valence and arousal state with the AS. Following this, an instruction about watching eight short videos was provided. Each of those was preceded by a video counter and followed by two questionnaires: the AS and the familiarity. The order of the videos and the order of the two sliders of AS were randomized over both parts of the experiments, fulfilling the condition that the labels of the videos are balanced. The first part of the experiment ended after watching eight videos and answering the corresponding questionnaire. The participant was allowed a short break after taking the EEG device and the headphones off.

**Figure 4:**
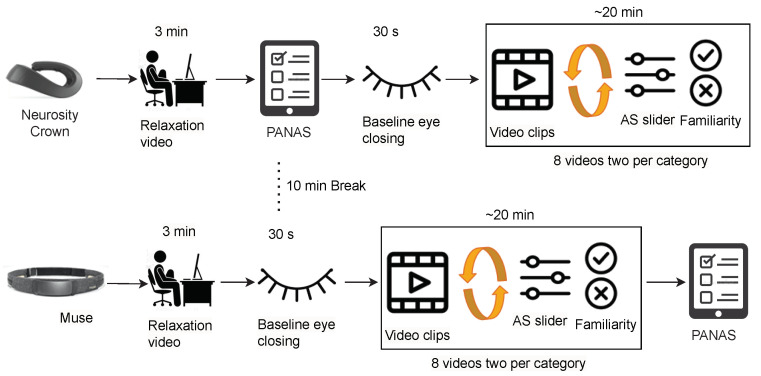
Experimental setup for curating Dataset II. The participants watched a relaxation video at the beginning and eight videos, two of each dimension category wearing one of the two devices. Between the eight videos, they answered AS slider and familiarity with the video.

In the second part of the experiment, the experimenter put the device that had not been used in the first part (Muse or Crown, respectively) and the headphones on the participant. Subsequently, the experimenter started the second part of the experiment, again after ensuring that the data collection was running smoothly. The participant followed the exact same protocol: watching the relaxation video, closing their eyes, and watching eight more movie scenes with the AS and familiarity questions in between. Lastly, they were asked for a final mood self-assessment via a second PANAS questionnaire to capture differences before and after the experiment. The experimental setup for curating Dataset II is depicted in Figure 4.

In this experiment, one PC (with a 2.4 to 3.0 GHz Dual Intel Core i5-6300U and 12 GB RAM) was used to present the stimuli and store EEG data to be used only after the experiment session.

### 2.7. Dataset III: Live Training and Classification

**Figure 5:**
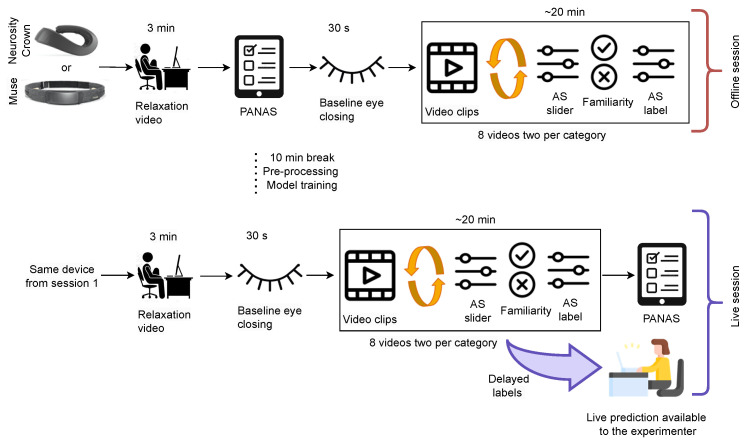
Experimental setup for curating Dataset III. In the first session, the participants watched a relaxation video at the beginning and eight videos, two of each dimension category wearing one of the two devices. Between the eight videos, they answered AS slider, familiarity with the video, and had seen the actual AS label. In the second session, they watched the same set of videos while the prediction was available to the experimenter before the delayed label arrived.

**Figure 6:**
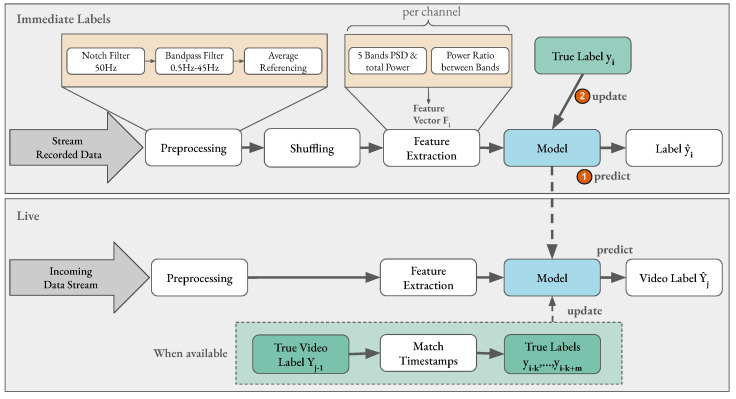
Overview of pipeline steps for affect classification. The top gray rectangle shows the pipeline steps employed in an immediate label setting with prerecorded data. For each extracted feature vector the model (1) first classifies its label before (2) being updated with the true label for that sample. In the live setting, the model is not updated after every prediction, as the true label of a video only becomes available after the stimulus has ended. The timestamp of the video is matched to the samples’ timestamps to find all samples that fall into the corresponding time frame and update the model with their true labels (shown in dotted lines).

The experimental setup for curating Dataset III is depicted in Figure 5. The participants received the same briefing as mentioned in Section 2.6. For both parts of the experiment, the same device was used. The protocol for the stimuli presentation in the first part (before the break) was the same as that as for the experiment with Dataset II, i.e., a relaxation video, PANAS, eye closing, eight video stimuli, and the AS and familiarity questions. One additional instruction after each AS was shown, which includes the original label of the videos. This additional information was given to the participant since the arousal ratings given in Dataset II were very imbalanced. During the break, the recorded EEG data were pre-processed and used to train an initial model in an online way. This initial model training was necessary because the data needed to be shuffled, as explained in Section 3.2. The initial model was continuously trained and updated during the second part of the experiment where a live prediction of affect is performed. The second part of the experiment was conducted similar to the first part: a relaxation video, eye closing, eight video stimuli, and the AS and familiarity questions. However, one additional prediction was performed and was available to the experimenter before the AS label from the participant. Furthermore, the AS label was used to update the model training and the prediction was running in parallel. Figure 6 displays the initialized model in the bottom gray rectangle that performed live emotion classification on the incoming EEG data stream. However, the prediction results were only displayed to the experimenter to avoid additional bias. Since the objective of this experiment was *live* online learning and classification, the data were coming in an online stream; however, the data were also stored for later evaluation and reproducibility.

In this experiment, the same PC from the previous experiment was again used to present the stimuli. Additionally, the AS label was sent to a second machine (MacBook Pro (2019) with a 2.8 GHz Quad-Core (Intel Core i7) and 16 GB). It also received EEG data, and performed data pre-processing, online model training, and live emotion classification.

## 3. Emotion Classification Pipeline

### 3.1. Data Pre-Processing

In this paper, the already pre-processed data from Dataset I were used, whereas Dataset II and Dataset III went through significant pre-processing to remove artifacts from the data [60,61]. Figure 6 depicts the pre-processing steps applied to both the immediate label setting (top) and in a live application (bottom). To remove the power-line interference visible on raw EEG recordings as a sinusoidal at 50 Hz (in Europe) [62], a second-order IIR notch digital filter was applied [63]. Furthermore, a fifth-order Butterworth band pass frequency filter from 0.5 to 45.0 Hz was applied to remove noise on irrelevant frequencies. Additionally, the data were average-referenced after filtering, i.e., the overall average potential was subtracted from each channel [22,37]. This method “relies on the statistical assumption that multichannel EEG recordings are uncorrelated” [64] and assumes an even potential distribution across the scalp. The pre-processing had to be minimal instead of using computation-heavy steps, since the live prediction had to be time efficient.

### 3.2. Data Windowing and Shuffling

**Figure 7:**
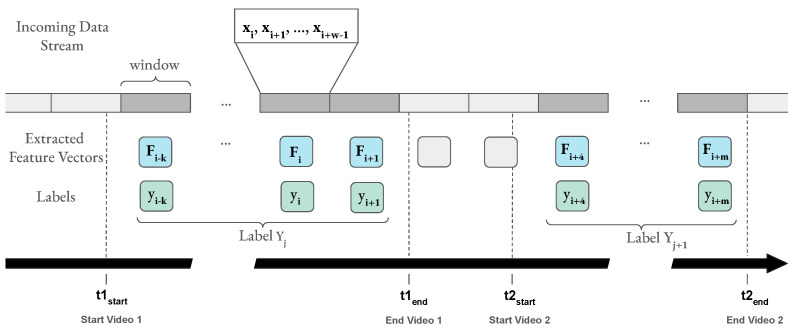
The incoming data stream is processed in tumbling windows (gray rectangles). One window includes all samples $x_{i},x_{i+1},\ldots$ arriving during a specified time period, e.g., 1 s. The pipeline extracts one feature vector, $F_{i}$, per window. Windows during a stimulus (video) are marked in dark gray. Participants rated each video with one label per affect dimension, $Y_{j}$. All feature vectors extracted from windows that fall into the time frame of a video (between $t_{start}$ and $t_{end}$ of that video) receive a label $y_{i}$ corresponding to the reported label, $Y_{j}$, of that video. If possible, the windows are aligned with the end of the stimulus; otherwise, all windows that lie completely inside a video’s time range are considered.

Since EEG data are considered stationary only over short time intervals, the pre-processing and the feature extraction were performed in tumbling windows with a fixed size and no overlap. Figure 7 shows that one window of the incoming data stream includes all samples $x_{i},x_{i+1},\ldots$ arriving during the specified window length. The pipeline extracts one feature vector, $F_{i}$, per window. All feature vectors extracted from the windows of a video duration (between $t_{start}$ and $t_{end}$) received a label $y_{i}$, corresponding to the reported label, $Y_{j}$, by the participants. Different window lengths, lϵ[1s,2s,3s,4s,5s], were tested on Dataset I and Dataset II to find the optimal one for the classification pipeline. As mentioned in the algorithm in Appendix A, a window, |w|, included *l* × sf samples with the sampling frequency denoted by sf.

Figure 7 shows that a lot of samples in a row received the same label of the duration of each video up to several minutes. Internal testing implied that training a model by streaming the data resulted in classifiers that did not learn from features but only returned the same class until seeing a different one. Therefore, the time windows were shuffled among one another with the corresponding labels. Since shuffling needs all data and labels to be present before feature extraction, it was not performed during live training and classification.

### 3.3. Feature Extraction

Similar to [22], power spectral density (PSD) features per channel were derived from the raw EEG data by using the Welch method [65] on each window. The PSD from each of the five frequency bands (Delta (0.5–4 Hz), Theta (4–8 Hz), Alpha (8–16 Hz), Beta (16–32 Hz), and Gamma (32–45 Hz)) and the total power over all frequency bands were extracted. Moreover, the power ratios between each pair of frequency bands were obtained. Therefore, a total of sixteen power-related features (five frequency bands + one total power + ten power ratios) were extracted from each channel, resulting in a different number of features per device as depicted in Table 2.

### 3.4. Labeling

During the live streaming of Dataset III, labels had to be mapped to their corresponding sample. Therefore, the labels were sent in a stream of tuples: $L_{1,\;A},L_{1,\;V},L_{2,\;A}$, $L_{2,\;V},\ldots$, where

(1) $$L_{j,\;dimension}=\left(Y_{j,\;dimension},\;t_{start},\;t_{end}\right)$$

*A* and *V* stand for arousal and valence, respectively, and $Y_{j,\;dimension}$ represents the AS label given by the participant after each video of two timestamps, $t_{start}$ and $t_{end}$. One labeled tuple $L_{j,\;dimension}$ per video and dimension was sent from the PC running the PsychoPy experiment to the PC training the classification model. The included timestamps were used to match the incoming ratings, $Y_{j,\;dimension}$, as labels to the samples that the model had classified before. This was performed in a way that all the samples that fell into the time period between $t_{start}$ and $t_{end}$ received the respective class label for each dimension. The model could then be updated with these labels.

### 3.5. Evaluation

**Figure 8:**
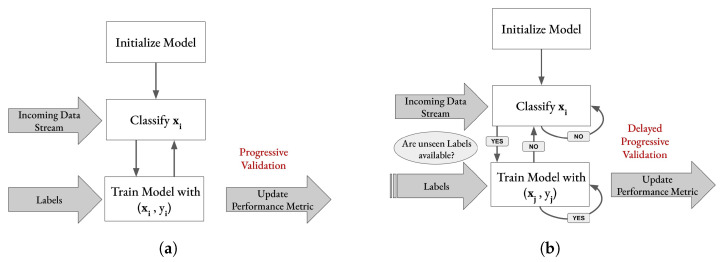
(**a**) Progressive validation incorporated into the basic flow of the training process (‘test-then-train’) of an online classifier in an immediate label setting. ($x_{i},y_{i})$ represents an input feature vector and its corresponding label. (**b**) Evaluation incorporated into the basic flow of the training process of an online classifier when labels arrive delayed (i≥j).

**Online Learning and Progressive Validation:** This paper aims at building a classification pipeline from evolving data streams. Therefore, the static data from Dataset I and Dataset II were streamed using a library for online learning: *river* [66]. *Progressive validation*, also called *test-then-train evaluation* [67], was used for model evaluation in the supervised immediate label setting, when the labels for all samples were present at processing time [68]. Figure 8a shows the training process of an online classifier including progressive validation. Every time the model sees a new sample $x_{i}$, it first classifies this sample as the test step of the test-then-train procedure. In the training process, the model will calculate the loss by comparing the true label, $y_{i}$, which might come from a different data source than the samples. The updated model will go on to classify the next incoming sample, $x_{i+1}$, before seeing its label, $y_{i+1}$, and, again, execute the training and performance metric updating step. This continues as long as data are streamed to the model. In this way, all samples can be used for training as well as for validation without corrupting the performance evaluation.

In the experimental setup acquiring Dataset III, the labels were available after the prediction, in contrast to the immediate labeling setting and progressive validation. Therefore, a *delayed progressive validation* was performed with the delayed labels, which is mostly the case for real-life scenarios. Figure 8b depicts the delayed progressive validation procedure, where the samples are classified by the model until unseen labels are available. However, the model can be updated as in the immediate label setting. Whenever new labels become available, the performance metric is updated without any further calculations [69]. Once the model has been updated with all available labels, the classification of further samples continues with the new updated model. This can, of course, be implemented in parallel as well. These steps continue as long as there are incoming data.

### 3.6. Machine Learning Classifiers

In this paper, three different algorithms, Adaptive Random Forest (ARF) [26], Streaming Random Patches (SRP) [70], and Logistic Regression (LR), have been evaluated and compared on Dataset I and Dataset II to find the best-performing setup for Dataset III. The ARF and the SRP with a Hoeffding Adaptive Tree (HAT) [42] are two ensemble architectures with integrated drift detection algorithms. Ensemble learners, which combine multiple weak learners, are popular in online learning not only because they tend to achieve high accuracy rates, but also because the individual learners of the ensemble can be trained in parallel. Furthermore, the structure of ensemble learners innately supports drift adaption, as drift detection algorithms can be easily incorporated and component learners can be reset [70,71]. The LR was included in the comparison as a sort of naïve baseline model by training on mini-batches (with partial fit) of one sample (i.e., a feature vector extracted from one window) to resemble the online learning process. Furthermore, it uses stochastic gradient descent for optimization with a learning rate of 0.1; no regularization was applied. For all models, the implementations from the river library [66] were used with default parameters if not specified otherwise.

**Evaluation Metrics:** The participants’ self-reported assessment of their valence and arousal levels was used as the ground truth in all training and evaluation processes in this paper. Among the different metrics of reporting the classifier’s performance [20], the commonly reported metrics Accuracy and F1-Score will be disclosed in this work. They are defined as follows [72]:
  (2) $$\begin{array}{cc} \mathrm{Accuracy} & =\frac{\mathrm{TP}+\mathrm{TN}}{\mathrm{TP}+\mathrm{TN}+\mathrm{FP}+\mathrm{FN}} \end{array}$$
  (3) $$\begin{array}{cc} F1\text{-}\mathrm{Score} & =\frac{\mathrm{TP}}{\mathrm{TP}+\frac{1}{2}\left(\mathrm{FP}+\mathrm{FN}\right)}, \end{array}$$
  where TP and TN denote the number of true positives and true negatives classified by the model, respectively. Accordingly, FP and FN stand for the number of false positives and false negatives classified by the model, respectively. “The higher, the better”, can be said for both accuracy and F1-Score, i.e., a perfect model has an accuracy of one (100%) and an F1-Score of one.

To determine whether the performance differences between the different setups were significant, two-sided t-tests with a significance level of α=0.05 were conducted on the respective dataset. When important, the results of these tests will be reported by either a p>0.05, meaning that no significant differences could be determined at this significance level, or by a p<0.05, denoting that the test showed the results of the two compared groups to be significantly different under this test setup.

## 4. Results

### 4.1. Immediate Label Setting

In this paper, at first the real-time emotion classification pipeline with immediate label setting was applied to Dataset I and Dataset II. The data were streamed to pre-process and to extract features from tumbling windows with a window length of 1 s. To perform binary classification for both dimensions of AS (valence and arousal), the self-rating of the participant was used by applying a threshold at 0.5 and defining *high* and *low* classes. As mentioned earlier, ARF, SRP, and LR classifiers were employed for evaluation. The setting of five trees and four trees for SRP worked best for Dataset I and for Dataset II, respectively. ARF included five trees for both data sets. A subject-dependent model was trained with 10-fold cross-validation, and the performances were evaluated with progressive validation.

**Figure 9:**
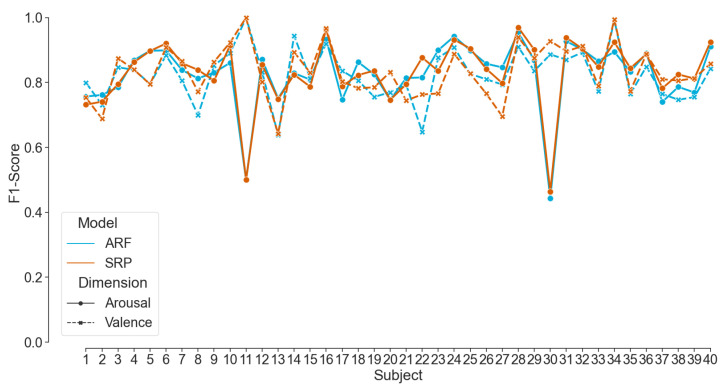
F1-Score for Valence and Arousal classification achieved by ARF and SRP per subject from Dataset I.

Figure 9 presents the overall model performances of Dataset I by showing the subject-wise distribution of the evaluation matrix. The mean F1-Score for the positive and negative classes of valence and arousal recognition, respectively, are shown only for ARF and SRP classifiers. The LR showed a comparatively poor performance since it is not an online model but trained on mini-batches. On the contrary, the ensemble models (ARF and SRP) show consistently higher F1-Scores, mostly between 0.7 and 0.95, with two outliers, which validates the emotion classification pipeline built in this paper. Two outliers are visible from subjects 11 and 30 and might be due to a label imbalance (high/low) in the data or insufficient data quality.

Furthermore, the means of F1-Score and accuracy over all the subjects from Dataset I are presented in Table 3. As depicted in “gray”, both evaluation matrices reach more than 80% for both the ensemble models (ARF and SRP), whereas the performance of LR is relatively poor. Additionally, Table 3 also shows the comparison to the evaluation of the baseline results by Miranda-Correa et al. [22] with a reported approximately 50% F1-Score with no accuracy score reported. Siddharth et al. [73] reports a more than 70% accuracy and F1-Score, and Topic et al. [74] achieved the current benchmark for this dataset by reporting a 90% accuracy. However, all the related work mentioned was obtained using a hold-out or k-fold cross-validation with computation-heavy offline classifiers, making it inadequate for real-time classification. Consequently, the proposed online classifiers with ensemble models can contribute to daily life setups.

Afterwards, we evaluated the pipeline using Dataset II curated by imitating a real-world setup. Table 4 presents the F1-Score of the subject-dependent models with the three classifiers for the positive and negative classes of arousal and valence recognition, respectively. The F1-Scores from both employed EEG devices are shown, with the best highlighted in bold. As depicted, all classifiers achieved higher performances on arousal recognition than on valence, which is in line with the literature [20,22]. Furthermore, the two ensemble methods ARF and SRP showed a better performance, with a mean F1-Score of more than 82% with no statistically significant difference (p>0.05) between them. LR models showed poor performance similar to their performance on Dataset I. Moreover, the mean F1-Score over all subject-dependent models using Crown data led to a better performance (by at least 2% and up to 7.6%) in most cases compared to using Muse data. However, the differences were not statistically significant (p>0.05) because four out of eleven cases for valence and five out of eleven cases for arousal recognition showed the F1-Score for this dataset. Thus, the distribution of which device’s data lead to the best performance is relatively balanced, leading to potential future work of analyzing specific electrode positions.

### 4.2. Effects of Window Size

**Figure 10:**
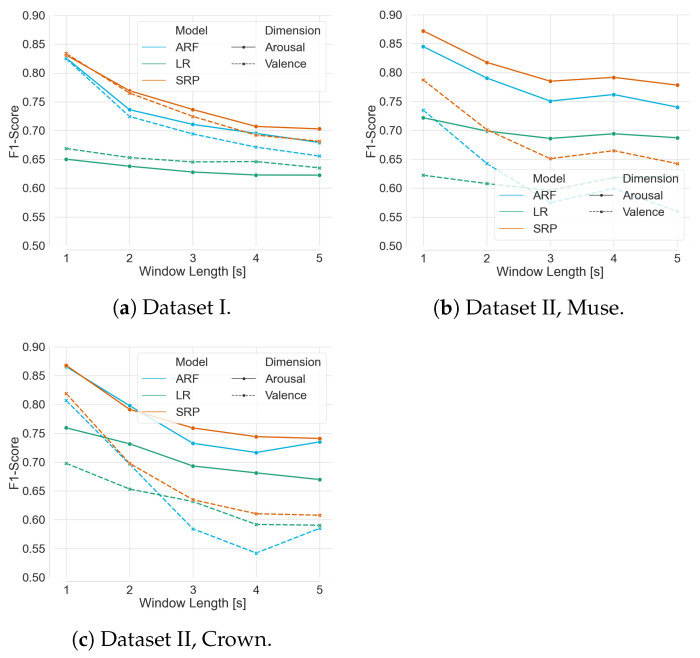
Mean F1-Score achieved by ARF, SRP, and LR over the whole dataset for both affect dimension with respect to window length.

As detailed in Section 3, the pipeline processes the incoming data and extracts the features that are used to train the model in tumbling windows of a specified length, lϵ[1s,2s,3s,4s,5s]. Using Dataset I and Dataset II (both Muse and Crown), the optimum window length was investigated for live prediction. As depicted in Figure 10, with 10-fold cross-validation and progressive validation, the mean F1-Scores from ARF and SRP classifiers show that the best classification performance was achieved with a window length of 1 s irrespective of the affect dimensions, classifiers, and devices. Moreover, in most cases, the classification performance decreases with increasing window size, emphasizing the need for more data points. Furthermore, these plots showcase again that the ensemble methods achieved overall higher F1-Scores than logistic regression and that all classifiers performed better on arousal recognition than on valence.

### 4.3. Delayed Label Setting: Live Classification

In order to validate the streaming setup of Dataset II, live predictions and live online training were performed while obtaining Dataset III. The participants wore the same EEG device for both parts of the experiment; participants 14 and 17 wore the Muse headband and participants 15 and 16 wore the Crown. For each participant, an ARF with four trees was trained on the data recorded in part 1 of the experiment using a window length of 1 s and progressive delayed validation. With the pre-trained model, live predictions were performed with the data streaming in part 2 of this experiment. The prediction results were only available to the experimenter and the model was continuously updated whenever new true labels became available from the participant. Therefore, the labels arrived with a certain delay depending on the length of the video. Table 5 shows that the highest F1-Score (in bold) obtained from each category during the live prediction was 73% for arousal and 60% for valence. However, most of the reported accuracies in Table 5 barely reached chance level.

**Figure 11:**
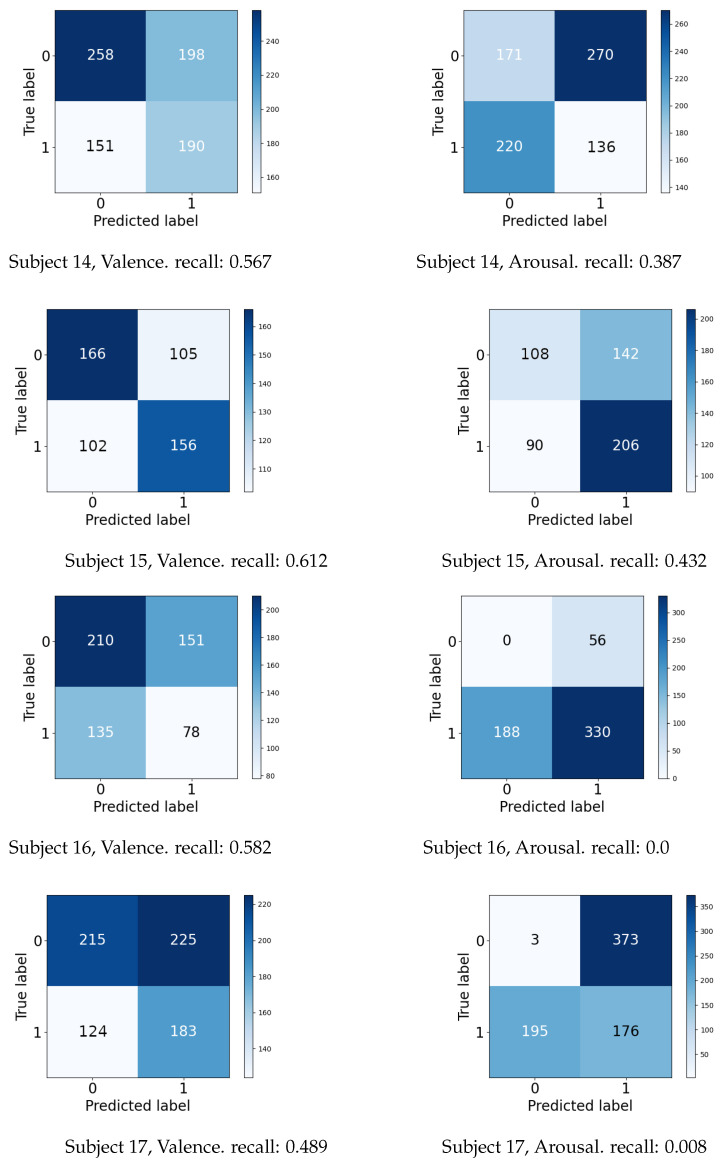
Confusion matrices for the live affect classification (Dataset III, part 2). Employed model: ARF (four trees), window length = 1 s. Recall was calculated only for a low class for both the models.

Furthermore, the prediction results are presented in the form of confusion matrices in Figure 11, showing that many of the samples have been misclassified. For three out of the four subjects, a low valence could be classified with a recall of at least 0.567, while for the arousal dimension, a low class was misclassified most often with a low recall. Though the proposed prediction pipeline was able to classify valence for over half the samples, it was unsuccessful in reliably predicting accurate classes for both affect dimensions. However, the findings from the live experiment show the importance of testing an application with settings as close as possible to the expected production scenario in order to get a reasonable performance estimate. Moreover, the results display the potential of emotion classification for live applications and motivate us to further investigate frameworks for real use cases with delayed labels instead of solely focusing on the immediate label setting.

Additionally, the lower predictive performance led us to further investigate the delayed labels. To imitate production settings, we induced a delay into the pipeline and applied progressive delayed validation on Dataset II. Therefore, the subject-dependent model was updated with the true label after 86 samples, i.e., the mean length of the video stimuli was 86 s. Table 6 displays the F1-Scores of both the models for valence and arousal recognition with a label delay of 86 s using an ARF with four trees and a window length of 1 s. The F1-Score for individual participants reached 77% for valence and 78% for arousal. However, the mean F1-Score across all participants achieved 63% for arousal and did not reach chance level for the valence classification. The performance declines significantly compared to Table 4, when a delay is induced.

However, the findings justify the poor performance in the live settings and validate the pipeline as a useful one with the possibility of modifications in future work. Furthermore, the binary arousal classification with the induced label delay outperforms the baseline results obtained by Miranda-Correa et al. [22] by 4.5% with an immediate label setting. However, the results reported by Siddharth et al. [73] and Topic et al. [74] outperform our work when used with immediate labels.

## 5. Discussion

In this paper, firstly, a real-time emotion classification pipeline was built for binary classification (high/low) of the two affect dimensions: *Valence* and *Arousal*. Adaptive random forest (ARF), streaming random patches (SRP), and logistic regression (LR) classifiers with 10-fold cross-validation were applied to the EEG data stream. The subject-dependent models were evaluated with progressive and delayed validation, respectively, when immediate and delayed labels were available. The pipeline was validated on existing data of ensured quality from the state-of-the-art AMIGOS [22] dataset. By streaming the recorded data to the pipeline, the mean F1-Scores were more than 80% for both ARF and SRP models. The results outperform the authors’ baseline results by approximately 25% and are also slightly better than the work reported in [73] using the same dataset. The results of Topic et al. [74] showed a better performance; however, due to the reported complex setup and computationally expensive methods, the system is unsuitable for real-time emotion classification. Nevertheless, the results mentioned in the related work apply offline classifiers with a hold-out or a k-fold cross-validation technique. In contrast, our pipeline applies an online classifier by employing progressive validation. To the best of our knowledge, no other work has tested and outperformed our online EEG-based emotion classification framework on the published AMIGOS dataset.

Secondly, a similar framework to the AMIGOS dataset, Dataset II, was established within this paper, which can collect neurophysiological data from a wide range of neurophysiological sensors. In this paper, two consumer-grade EEG devices were used to collect data from 15 participants while watching 16 emotional videos. The framework available in the mentioned repository can be adapted for similar experiments.

Thirdly, and most importantly, we curated data in two experiments to validate our classification pipeline using the mentioned framework. Eleven participants took part in acquiring th data for Dataset II, where EEG data were recorded while watching 16 emotion elicitation videos. The pre-recorded data were streamed to the pipeline and showed a mean F1-Score of more than 82% with ARF and SRP classifiers using progressive validation. This finding validates the competence of the pipeline on the challenging dataset from consumer-grade EEG devices. Additionally, the online classifiers consistently showed better performance for ARF and SRP than LR on all compared modalities. However, internal testing verified that the run-time of the training step of the pipeline of ARF is less than that of SRP, concluding that ARF should be used in live prediction. The analysis on window length shows a clear trend of increasing performance scores with decreasing window length; therefore, a window length of 1 s was chosen for live prediction. Although the two employed consumer-grade devices have a different number of sensors at contrasting positions, there were no statistically significant differences between the achieved performance scores found for their respective data. Therefore, we used both devices for live prediction, and the pipeline was applied to a live incoming data stream in the experiment of Dataset III with the above-mentioned features of the model. In the first part of the experiment, the model was trained with the immediate labels from the EEG data stream. In the second part, the model was used to predict affect dimensions while the labels were available after a delay of the video length. The model was continuously updated whenever a new label is available. The performance scores achieved during the live classification with delayed labels were much lower than those with immediate labels, motivating us to induce an artificial delay to stream Dataset II. The results are compatible with live prediction. The literature reports better results for real-time emotion classification frameworks [29,30,36] with the assumption of knowing the true label immediately after a prediction. The novelty of this paper is to present a real-time emotion classification pipeline close to a realistic production scenario from daily life with the possibility of including further modifications in future work.

In future work, the selected stimuli can be shortened to reduce the delay of the incoming labels so that the model can be updated more frequently. Otherwise, multiple intermediate labels can also be included in the study design to ensure the inclusion of short-term emotions felt while watching the movies. Furthermore, more dynamic pre-processing of the data can be included with feature selection algorithms for better classification and prediction in live settings. Moreover, the collected data from the experiments reveal a strong class imbalance in the self-reported affect ratings for arousal, with high arousal ratings making up 82.96% of all ratings in that dimension. This general trend towards higher arousal ratings is also visible in Dataset I, albeit not as intensely (62.5% high arousal ratings). In contrast, Betella et al. [59] found “a general desensitization towards highly arousing content” in participants. The underrepresented class can be up-sampled in the model training in the future, or basic emotions can be classified instead of arousal and valance dimensions, solving the multi-class problem [75,76]. By including more participants in the future for live prediction, the prediction can be visible to the participant as well to include neuro-feedback. It will also be interesting to see if the predictive performance improves by utilizing additional modalities other than EEG, for example, heart rate and electrodermal activity [20,23,37].

## Figures and Tables

**Table 1:** The source movies of the videos used are listed per quadrant in the valence–arousal space. Video IDs are stated in parentheses, sources marked with a † were taken from the MAHNOB-HCI dataset [55]; all the others stem from DECAF [54]. In the category column, H, L, A, and V stand for high, low, arousal, and valence, respectively. This table has been adapted from Miranda-Correa et al. [22].

Category	Source Movie
HAHV	*Airplane* (4), *When Harry Met Sally* (5), *Hot Shots* (9), *Love Actually* (80)${}^{†}$
LAHV	*August Rush* (10), *Love Actually* (13), *House of Flying Daggers* (18),
	*Mr Beans’ Holiday* (58)${}^{†}$
LALV	*Gandhi* (19), *My Girl*(20), *My Bodyguard* (23), *The Thin Red Line* (138)${}^{†}$
HALV	*Silent Hill* (30)${}^{†}$, *Prestige* (31), *Pink Flamingos* (34), *Black Swan* (36)

**Table 2:** Number of channels and derived features for each device: Muse Headband: 64 features; Neurosity Crown: 128 features; Emotiv EPOC: 224 features.

Device	Number of Channels	Number of Derived Features
Muse Headband	4	64
Neurosity Crown	8	128
Emotiv EPOC	14	224

**Table 3:** Comparison of mean F1-Scores and accuracy of Valence and Arousal recognition on Dataset I. Gray represents the results from this paper. NR stands for not reported.

Study or Classifier	F1-Score		Accuracy	
	Valence	Arousal	Valence	Arousal
LR	0.669	0.65	0.702	0.688
ARF	0.825	0.826	0.82	0.846
SRP	0.834	0.831	0.826	0.847
Miranda-Correa et al. [22]	0.576	0.592	NR	NR
Siddharth et al. [73]	0.8	0.74	0.83	0.791
Topic et al. [74]	NR	NR	0.874	0.905

**Table 4:** Comparison of mean F1-Scores of Arousal and Valence recognition per participant and device from Dataset I with three classifiers using progressive validation. Bold values indicate the best-performing model per participant and dimension. The mean total represents the calculated average of all models’ F1-Scores.

Subject ID		ARF		SRP		LR	
		Crown	Muse	Crown	Muse	Crown	Muse
Arousal	3	**0.902**	0.885	0.895	0.898	0.8	0.785
	4	0.836	0.794	0.838	**0.845**	0.793	0.604
	5	0.651	0.812	0.699	**0.827**	0.764	0.682
	6	0.836	0.843	0.863	**0.889**	0.771	0.62
	7	**0.958**	0.833	0.933	0.878	0.841	0.725
	8	0.889	0.749	**0.893**	0.783	0.683	0.584
	9	0.888	0.921	0.836	**0.931**	0.756	0.703
	10	**0.969**	0.903	0.951	0.915	0.816	0.898
	11	0.938	0.768	**0.955**	0.861	0.765	0.908
	12	0.864	0.871	**0.884**	0.878	0.669	0.697
	13	0.792	**0.913**	0.8	0.887	0.701	0.734
	Mean	0.866	0.845	0.868	**0.872**	0.76	0.722
Valence	3	0.837	**0.887**	0.811	0.876	0.716	0.712
	4	0.841	0.69	0.773	**0.859**	0.804	0.524
	5	0.546	0.734	0.639	0.748	**0.781**	0.58
	6	0.713	0.687	**0.785**	0.778	0.73	0.393
	7	**0.935**	0.666	0.926	0.757	0.776	0.616
	8	0.813	0.551	**0.819**	0.623	0.594	0.444
	9	0.812	0.844	0.721	**0.863**	0.72	0.561
	10	**0.982**	0.859	0.979	0.871	0.74	0.874
	11	0.924	0.653	**0.957**	0.811	0.64	0.884
	12	0.889	0.756	**0.914**	0.784	0.633	0.663
	13	0.584	**0.826**	0.6	0.775	0.543	0.595
	Mean	0.807	0.735	**0.819**	0.787	0.698	0.622

**Table 5:** F1-Score and accuracy for the live affect classification (Dataset III, part 2). Subjects 14 and 17 wore Muse, while subjects 15 and 16 wore the Crown for data collection. The highest scores across all participants for each evaluation matrix are marked in bold.

Subject ID	F1-Score		Accuracy	
	Valence	Arousal	Valence	Arousal
14	0.521	0.357	0.562	0.385
15	**0.601**	0.64	**0.609**	**0.575**
16	0.353	**0.73**	0.502	**0.575**
17	0.512	0.383	0.533	0.24

**Table 6:** Mean F1-Scores for Valence and Arousal recognition of Dataset II, relayed per participant and device. Obtained using ARF (with four trees), a window length of 1 s, and progressive delayed validation with a label delay of 86 s. The bold values represent the highest scores, and the last row shows the mean F1-Score of all participants.

Participant ID	Valence		Arousal	
	Crown	Muse	Crown	Muse
3	0.338	0.584	0.614	0.718
4	0.674	0.429	0.551	0.575
5	0.282	0.554	0.355	0.69
6	0.357	0.27	0.608	0.619
7	0.568	0.574	0.698	0.769
8	0.266	0.286	0.561	0.574
9	0.553	0.53	0.719	0.749
10	**0.767**	0.561	**0.784**	0.691
11	0.469	0.207	0.676	0.418
12	0.443	0.51	0.575	0.679
13	0.335	0.451	0.646	0.711
Mean	0.476	0.46	0.637	0.637

## Data Availability

The dataset is available from the authors upon request for scientific purposes at https://doi.org/10.5281/zenodo.7398263 (accessed on 18 February 2023). The source code used for analysis in this study can be found at https://github.com/HPI-CH/EEGEMO (accessed on 18 February 2023).

## Abbreviations

The following abbreviations are used in this manuscript:

MDPI	Multidisciplinary Digital Publishing Institute
ARF	Adaptive Random Forest
AS	Affective Slider
EEG	Electroencephalography
HCI	Human–Computer Interaction
HVLA	High Valence Low Arousal (different combinations are possible)
LR	Logistic Regression
OSC	Open Sound Control
PANAS	Positive Furthermore, Negative Affect Schedules
PSD	Power Spectral Density
SRP	Streaming Random Patches

## Appendix A

**Algorithm A1:** Live Emotion Classification from an EEG Stream.
